# The First Successful Case of Transoral Robotic Surgery in a Patient with Sialadenoma Papilliferum

**Published:** 2016-09

**Authors:** Arzu-Karaman Koç, Yakup Yegin, Mustafa Çelik, Mehmet Sar, Damlanur Sakiz, Fatma-Tülin Kayhan

**Affiliations:** 1 *Department of Otorhinolaryngology - Head and Neck Surgery, Bakırköy Dr.Sadi Konuk Training and Research Hospital, Istanbul, Turkey.*; 2 *Department of Pathology,Bakırköy Dr.Sadi Konuk Training and Research Hospital, Istanbul, Turkey.*

**Keywords:** Minor salivary gland, retromolar pads, Sialadenoma papilliferum, Transoral robotic surgery

## Abstract

**Introduction:**

Sialadenoma papilliferum (SP) is a rare benign tumor, which originates from the minor salivary gland. It occurs at sites that have minor salivary glands, such as the palate, retromolar pads, buccal mucosa, and lips. The most common location for tumor development is on the hard palate. A differential diagnosis consists of ruling out other salivary gland tumors. Transoral robotic surgery (TORS) is a new technology used in head and neck surgery within certain centers around the world.

**Case Report:**

Herein, we present the first successful case of SP tumor removal by TORS.

**Conclusion:**

This particular case highlights the identification of this rare tumor in an unusual location. Furthermore, it demonstrates the utilization of TORS, leveraging the superior visualization to obtain excellent local control with minimal acute and long-term morbidity, in comparison to conventional transoral surgical approaches.

## Introduction

First described by Abrams and Finck in 1969, Sialadenoma papilliferum (SP) is a rare benign tumor that often occurs at sites with minor salivary glands (1). The tumor constitutes less than 1% of minor salivary gland tumors, despite the potential to occur at every site in the mouth with a minor salivary gland. The most common location for these tumors, as reported in the literature, is on the hard palate (1,2). Cases of retromolar pads are rarely reported. The typical treatment for SP is complete excision with clear surgical margins. However, there are currently several surgical approaches, each contingent on tumor location. In literature, only 57 cases of SP have been reported between 1969 and 2015. This case was the fifty-eighth case of SP in literature. Although current literature knowledges modify due to case reports, the gold standart method of treatment has not yet been determined. Even though many different surgical approaches were described in literature, TORS that is a new technology used in head and neck surgery within certain centers around the world was used in this case. Considering current literature, the first case of a patient with SP located on the retromolar pad, which was successfully treated by TORS was reported. Herein, this case report highlights the novel use of TORS for excision of this rare case of SP.

## Case Report

**Fig 1:**
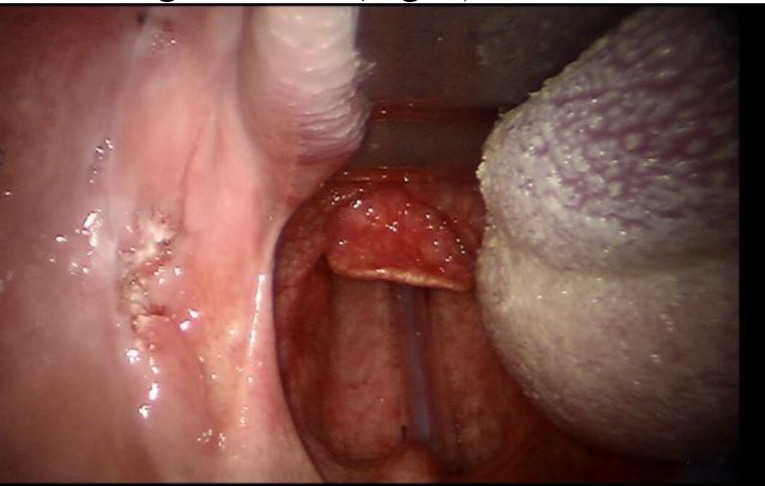
The view of oropharynx examination

A 72-year-old male smoker presented with progressive enlargement of a painful mass extending from the buccal mucosa to the mandıble corpus on his left retromolar pad. The mass had been growing for over 1 year and caused occasional bleeding. The patient’s history indicated a lack of any surgeries or chronic infection, but a history of smoking 65 packets of cigarettes per year. Oropharyngeal examination revealed the presence of an irregular, exophytic lesion in the left retromolar pad measuring approximately 3x3 cm, with a papillamatous hemorrhagic surface (Fig. 1). 

**Fig 2:**
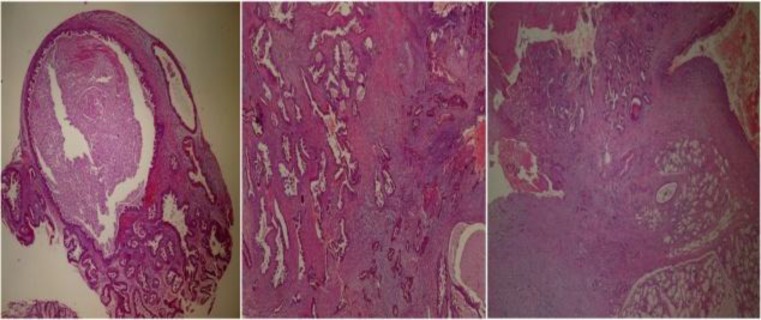
Microscopic view of sialadenoma papilliferum: Papillary structures within cystic glandular spaces (5x, hemotoxylin-eosin stain

Further otolaryngological and systemic examinations were unremarkable. Complete blood count (CBC), biochemistry, and coagulometer tests were applied. Incisional biopsy was performed because the patient was suspected of having a malignant salivary gland tumor. Computerized tomography (CT) and magnetic resonance imaging (MRI) was performed to reveal the relationship between bone structure and soft tissue within the oral cavity. All of the hematological test results were unremarkable. Histopathologically biopsy specimen showed dilated duct-like structures with an overliying squamous epithelium. The duct like structures were lined by double-triple layered epithelium with prominent papillary proliferations. The papillae showed thin fibrovascular cores. The result of the biopsy was consistent with SP (Fig. 2). 

**Fig 3:**
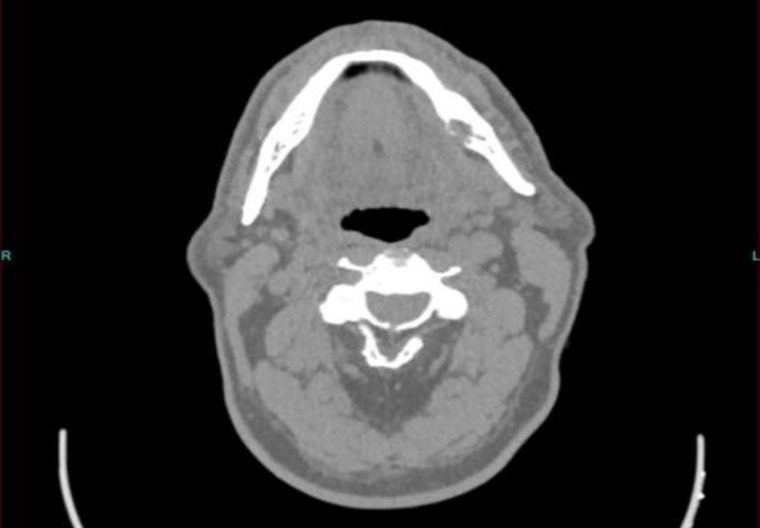
Axial view of CT images: a lytic lesion in the left mandibular corpus

CT revealed the presence of a lytic lesion in the left mandibular corpus measuring approximately 19 mm (Fig.3). MRI revealed the presence of some reactive lymphadeno- pathy . Complete excision of the mass was suggested, and sparing patient from highly morbid external surgical procedures with longer hospital stays and increased complications, the option of TORS was offered to the patient.

The advantages and disavantages of TORS were explained to the patient. 

The patient chose to proceed with TORS, and subsequently underwent tumor excision via TORS with a laser-resistant nasotracheal tube under general anesthesia. The da Vinci robotic system was set up and docked with the patient cart. A Feyh-Kastenbauer retractor was used to expose the oropharynx. A 8 mm 0-degree da Vinci three-dimensional robotic camera was introduced into the oral cavity to visualize the operating field.

Then two lateral arms were attached. For the TORS-SP, a 5 mm monopolar cautery device was attached to one arm, and a Maryland dissector was attached to the second arm. The tumor was completely removed with a 1 cm clear surgical margin, using a monopolar cautery device.

**Fig 4:**
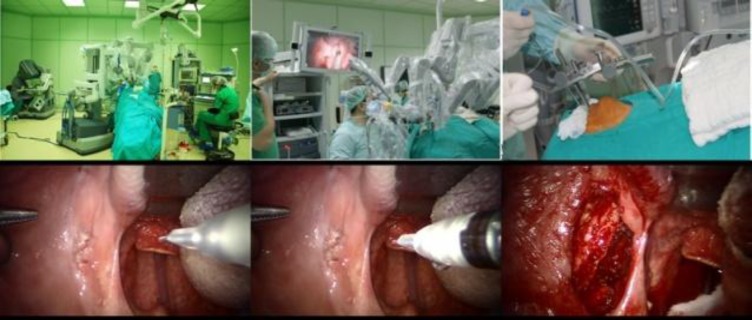
The view of surgical fields and steps

The lytic lesion in left mandibular corpus was curetted completely and the defect in left mandibular corpus was primarily covered with oral mucosa. The Maryland dissector was replaced with a needle-driver, and surgical closure was performed using 2-0 vicryl (Fig.4). 

The total operation time was 30 min including a 5 min robot setup time. Total blood loss was limited to 10 cc during surgery. The patient started peroral liquid intake six h after the operation. Histopathologicaly the diagnosis was SP in the excision specimen. The result of curetted lytic bone lesion was consistent with SP. Postoperative complications and recurrence were not observed during the 1-year follow-up. 

***Informed Consent***

Written informed consent was obtained from the patient described in this case. 

## Discussion

First described by Abrams and Finck in 1969, SP is an extremely rare benign tumor of the minor salivary gland (1). It presents more frequently in males, and can occur at any age (2,3). According to the current literature, the most common locations for SP tumors are the hard palate, buccal mucosa, lips, and retromolar pads (3,4). Theoretically, SP tumors can occur at any site with minor salivary glands. However, only two cases have been reported on the base of the tongue, which has more minor salivary glands than any other location (5). Other uncommon locations for SP include the parotid gland of which four cases were previously reported (2,4).

SP is a benign, exophytic, slow-growing, painless tumor of unknown origin. In the literature, there is inconstancy between the time the tumor is first noticed by the patient and when it is presented to the physician. The patient described in the study by Abrams and Finck (1,4) had the tumor for 10 to 12 years prior to presentation. In our case, the tumor was present for 1 year prior to presentation. 

SP is diagnosed by a correlation between clinical indications and histopathological examination. Microscopically, SP is characterized by an exophytic mass without encapsulation, and papillary proliferation of salivary gland ductal epithelium. This typically includes a double-layer of polyhedral and columnar cells with a concomitant overgrowth of squamous epithelium. A differential diagnosis of SP consists of eliminating the potential for several benign and malign salivary gland tumors such as mucoepidermoid carsinoma, papillary cystadenoma, adenolymphema, intraductal papillary tumors, Warthin’s tumor, and papillary squamous cell carcinoma (4,5). Therefore, clinical indications, differential diagnosis, and histopathological evaluation are very important for the diagnosis of SP. Although SP is a benign tumor, surrounding tissue can be affected contingent on the growth pattern. Despite some reported cases of skin ulceration causing a exophytic fungating growth pattern, no lytic bone lesions have been reported (4). In our case, a lytic defect on mandibuler corpus was present, which was the first case in the literature.

Treatment of SP involves local excision of the tumor, and many different surgical approaches currently exist (5,6). In our case, the patient underwent tumor excision via TORS, which has not been previously described in the literature. To the best of our knowledge, this is the first case to use TORS for SP removal from the retromolar pad. TORS, which is also used in head and neck surgery in certain centers around the world, is a very new technique. It was first described by Weinstein and O’Malley in 2005, and has subsequently been used successfully for supraglottic partial laryngectomy, radical tonsillectomy, tongue base resection, and vocal cord surgery. 

In January 2010, the Food and Drug Administration approved TORS for use in benign and malignant disease of the tonsils, pharynx, and larynx (7). The use of TORS has since been described for cordectomy, praxis of nasopharynx, and oropharynx (8-10). In our clinic, we have successfully used TORS in over 150 operations since 2010. TORS has several benefits, such as limited surgical morbidity, mortality, shorter hospital stay, good hemostasis, less pain, and postoperative maintenance of quality of life (7,8). The disadvantage of TORS is the relative expense of the procedure compared to other available options, and its novelty means that there are still a number of uncertainties associated with its use (7).

In addition to the rarity of and little knowledge about this neoplasm, this case report exemplifies how TORS can be employed for excellent local control with minimal morbidity. The lytic bone lesion in SP of oral cavity is not contraindication for TORS. However, there is no enough knowledge about bone involvement and TORS for SP in literature.

The prognosis of SP is excellent, and recurrence is not expected. Malignant transformation is very rare, but in literature some cases of mucoepidermoid carcinoma arising in a background of SP have been reported (5,6). Close follow-up with the patient is important to reduce the risk of malignant transformation. 

## Conclusions

This case presented here highlights the identification of this rare tumor in an unusual location. Furthermore, it demonstrates the utilization of TORS, leveraging the superior visualization to obtain excellent local control with minimal acute and long-term morbidity in comparison to conventional transoral surgical approaches. 
